# Toward a full-scale computational model of the rat dentate gyrus

**DOI:** 10.3389/fncir.2012.00083

**Published:** 2012-11-16

**Authors:** Calvin J. Schneider, Marianne Bezaire, Ivan Soltesz

**Affiliations:** Department of Anatomy and Neurobiology, University of California IrvineIrvine, CA, USA

**Keywords:** computational model, dentate gyrus, parallel, morphology, variability

## Abstract

Recent advances in parallel computing, including the creation of the parallel version of the NEURON simulation environment, have allowed for a previously unattainable level of complexity and detail in neural network models. Previously, we published a functional NEURON model of the rat dentate gyrus with over 50,000 biophysically realistic, multicompartmental neurons, but network simulations could only utilize a single processor. By converting the model to take advantage of parallel NEURON, we are now able to utilize greater computational resources and are able to simulate the full-scale dentate gyrus, containing over a million neurons. This has eliminated the previous necessity for scaling adjustments and allowed for a more direct comparison to experimental techniques and results. The translation to parallel computing has provided a superlinear speedup of computation time and dramatically increased the overall computer memory available to the model. The incorporation of additional computational resources has allowed for more detail and elements to be included in the model, bringing the model closer to a more complete and accurate representation of the biological dentate gyrus. As an example of a major step toward an increasingly accurate representation of the biological dentate gyrus, we discuss the incorporation of realistic granule cell dendrites into the model. Our previous model contained simplified, two-dimensional dendritic morphologies that were identical for neurons of the same class. Using the software tools L-Neuron and L-Measure, we are able to introduce cell-to-cell variability by generating detailed, three-dimensional granule cell morphologies that are based on biological reconstructions. Through these and other improvements, we aim to construct a more complete full-scale model of the rat dentate gyrus, to provide a better tool to delineate the functional role of cell types within the dentate gyrus and their pathological changes observed in epilepsy.

## Introduction

The dentate gyrus network model was developed to study the role of the circuit alterations present in the epileptic dentate gyrus (Santhakumar et al., 2005; Dyhrfjeld-Johnsen et al., 2007) and has been used to make predictions for the existence of non-random microcircuits in epilepsy (Morgan and Soltesz, 2008). The network model has also been used in other studies on topics such as epilepsy (Thomas et al., 2009, 2010), paired-pulse inhibition (Jedlicka et al., 2010), excitability (Winkels et al., 2009; Jedlicka et al., 2011), computational modeling software (Gleeson et al., 2007), and in the construction of a CA1 model (Cutsuridis et al., 2010). Of particular note is the use of the model to test improvements to the NEURON simulation environment (Migliore et al., 2006; Hines and Carnevale, 2008; Hines et al., 2008a,b). The network simulations performed in the studies listed above have exclusively used the smaller 1:2000 scale model (Santhakumar et al., 2005), in part due to the increased time associated with simulating the 1:20 scale model, with a reported 35–70 h per simulation (Dyhrfjeld-Johnsen et al., 2007).

The creation of parallel NEURON and the speedup obtained during tests on the 1:2000 scale model (Migliore et al., 2006) have provided a means to not only make the recent model more accessible to researchers, but to also remove the limitations on the size and complexity of the computational model. The results presented in this study represent the next step in model development, expanding the size and scope of the model. The results from the translation of the 1:20 scale model from a serial to parallel implementation are described, as well as the enlargement of the model to the full size of the rat dentate gyrus. Through the cooperation of two software tools, the complexity of the model can be increased with the generation of variable and realistic dendritic morphology for the sample case of granule cells. These model improvements reflect a significant advancement toward a realistic full-scale computational model.

## Materials and methods

All simulations, virtual neuron generation, and analysis were performed on the UCI Broadcom Distributed Unified Cluster (BDUC) or a PC running Ubuntu Linux. Data analysis and plotting were performed using Python 2.7.1.

### Dentate gyrus network simulations

All network simulations were performed using NEURON 7.0 (Hines and Carnevale, 1997). NEURON was configured to run in parallel on the Linux BDUC cluster with Openmpi 1.4.3, as shown previously (Hines and Carnevale, 2008). The serial version of the 1:20 scale model is freely available at ModelDB (http://senselab.med.yale.edu/modeldb/ShowModel.asp?model=124513). The serial version of the dentate model was translated to a parallel implementation using strategies described elsewhere (Migliore et al., 2006; Hines and Carnevale, 2008). This involved assigning each cell a global identifier, so that even though a cell is only created on one host, all other hosts are still be able to refer to that cell. Each cell was then associated with its own random number generator for creating connections which uses a seed that is dependent on its global identifier. The dentate model NEURON code has also been rewritten with an emphasis on modularization and explanatory commenting in order to increase its adaptability and accessibility for the general neuroscience community.

The 1:20 scale parallel model was constructed in accordance with the serial model from previous studies (Dyhrfjeld-Johnsen et al., 2007; Morgan and Soltesz, 2008). Multicompartmental models for granule cells, mossy cells, basket cells, and hilar cells with axonal projections to the perforant path (HIPP) were taken from the original 1:2000 scale model (Santhakumar et al., 2005). These are the numerically dominant cell types found in the dentate gyrus. The single-cell models contained ionic currents dependent on the given cell type, including sodium, fast- and slow-delayed rectifier potassium, A-type potassium, I_h_, L, N, and T-type calcium, and calcium-dependent potassium currents. The ionic currents in the cell models were previously tuned to match the passive and active properties observed experimentally for each cell type (Santhakumar et al., 2005). Connectivity was based on a highly realistic, data-driven structural model (Dyhrfjeld-Johnsen et al., 2007), and the probability of making a connection between two cells was increased fivefold to account for the reduced size of the network. The two elements of the injured model (hilar cell loss and mossy fiber sprouting) were simulated at 80% of their maximum values, as this value produced maximal epileptiform activity in the network model and corresponds to the level of mossy cell survival observed in human temporal lobe epilepsy (Blumcke et al., 2000; Gabriel et al., 2004). Perforant path stimulation was simulated through simultaneous input to 5000 granule cells (10%), 10 mossy cells (3.3%), and 50 basket cells (10%) located in the middle lamella of the model dentate gyrus at 5 ms from the start of the simulation.

The full-scale model eliminated previous scaling adjustments that were required by the smaller network size. The number of cells was the same as estimated to be in the dentate gyrus in the rat (for a detailed description, see Dyhrfjeld-Johnsen et al., 2007), with 1,000,000 granule cells, 30,000 mossy cells, 10,000 basket cells, and 12,000 HIPP cells evenly distributed along the septotemporal axis. Hilar cell loss and mossy fiber sprouting were again simulated at 80% of their maximum. The peak conductance for sprouted granule cell synapses was reduced from 1.0 to 0.5 nS, the original estimate based on experimental data (Molnar and Nadler, 1999). Synaptic conductances for connections from granule cells to both inhibitory cell types were reduced by a factor of two to avoid depolarization block, as done previously (Dyhrfjeld-Johnsen et al., 2007). Perforant path stimulation was simulated through simultaneous input to 10,000 granule cells (1%), 20 mossy cells (0.33%), and 100 basket cells (1%), which corresponds to stimulation of 1/10th of the middle lamella of the model dentate gyrus.

### Reconstructions

Digital reconstructions of dendritic trees were obtained from 19 granule cells labeled *in vivo* in the rat dentate gyrus (Buckmaster, 2012). The three-dimensional Neurolucida reconstructions were corrected for shrinkage in the transverse (1.06X) and depth (1.96X) planes based on previous estimates (Buckmaster and Dudek, 1999) using Neurolucida software (MicroBrightfield, Williston, VT). Reconstruction files were converted from.DAT to.ASC format for compatibility with morphological analysis.

### Analysis and generation of dendritic morphology

The creation of realistic dendritic morphologies was performed using two freely available software programs: L-Measure and L-Neuron. Morphological parameters from granule cell reconstructions and generated virtual neurons were extracted using L-Measure v4.0 software (Scorcioni et al., 2008). L-Measure is available at http://cng.gmu.edu:8080/Lm/. Virtual dendritic trees were generated using L-Neuron v1.08 (Ascoli and Krichmar, 2000), available at http://krasnow1.gmu.edu/cn3/L-Neuron/index.htm. The L-Neuron program was executed with the Hillman/PK dendritic growth algorithm (Ascoli et al., 2001), and outputs were generated in Southampton Archive format (.swc) for compatibility with L-Measure analysis.

The parameters utilized by the dendritic growth algorithm, referred to as basic parameters, were extracted from the granule cell reconstructions as raw values. Extracted basic parameter distributions were then incorporated into L-Neuron using one or more of the statistical distributions allowed in L-Neuron: gamma, normal, uniform, and constant value distributions. Many of the basic parameters are compatible and can thus be directly incorporated into L-Neuron from L-Measure. The definitions for some basic parameters, however, are different between L-Neuron and L-Measure, which can be modified to create congruency. For example, L-Neuron generates terminal dendritic branches by creating an interbifurcation segment and then attaching a terminal segment, whereas L-Measure analyzes the two segments as a single branch. This creates a discrepancy between the distribution input for L-Neuron and the distribution for generated outputs measured with L-Measure for parameters such as the path length of the terminal branch. Adjustments were made to the L-Neuron input to maximize the overlap between the basic parameter distributions extracted from generated virtual neurons and those extracted from reconstructions. As a result, L-Neuron creates morphologies that have similar basic parameters, including the path length of terminal branches noted above, as the sample reconstructions. For statistical tests, the outputs for the constant value distributions in L-Neuron were set to their intended values due to minor deviations imposed in the L-Neuron program.

Morphological parameters not used in the dendritic growth algorithm, known as emergent parameters, were used to compare virtual and real neurons, as well as to filter for biologically realistic virtual granule cells. Scalar emergent parameters summarize a morphological characteristic in a single value, whereas distribution emergent parameters show the dependence of one parameter on another. The scalar and distribution emergent parameters used in this study were largely taken from a previous study using L-Neuron (Ascoli et al., 2001). The scalar emergent parameters used were total dendritic length, number of bifurcations, surface area, average path distance to dendritic tips, average Euclidean distance to tips, maximum Euclidean distance to tips, maximum branch order, partition asymmetry, transverse spread, and longitudinal spread. The transverse and longitudinal spreads were used instead of height, width, and depth in order to provide an orientation-independent measure of the three-dimensional extent of dendritic trees. Transverse spread was defined as the maximum distance in the xy plane between dendritic tips, while longitudinal spread was the maximum distance in the z plane. Generated virtual neurons were selected if they fell within two standard deviations of the mean for all emergent parameters except surface area. Assuming a normal distribution, this theoretically includes more than 95% of the granule cell population. The two standard deviation limit for surface area would have allowed for unrealistic values that were lower than the allowed length, so the constraint was changed to 1.65 standard deviations (theoretically more than 90% of granule cells).

## Results

### Parallelization of the 1:20 scale model

**Figure 1:**
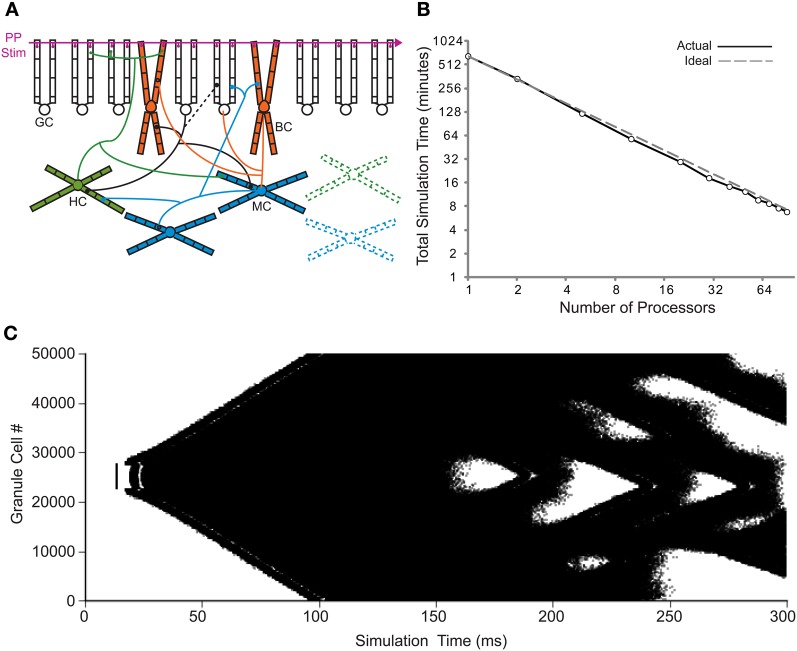
**Translation to parallel NEURON. (A)** Basic network connectivity of the dentate model. A depiction of the dendritic structure, connectivity, and location of synapses for the four cell types is shown. Note that the septo-temporal extent of the axons is also incorporated into the model (see Dyhrfjeld-Johnsen et al., 2007) but is not illustrated here. In the injured model, HIPP cells and mossy cells are lost, while granule cells synapse onto other granule cells (changes are represented by dashed lines). GC, granule cell; BC, basket cell; HC, HIPP cell; MC, mossy cell; and PP Stim, perforant path stimulation. **(B)** Runtime of 80% injured network simulations versus the number of processors for actual and ideal scaling. **(C)** Granule cell activity for the parallel 1:20 scale model. Each dot represents a single granule cell spike.

Previous implementations of the rat dentate gyrus network model were scaled down from the biological dentate gyrus because they were limited to the use of a single processor. The creation of parallel NEURON (Migliore et al., 2006) dramatically increased the computational resources available to network simulations. Increasing the number of processors for the 1:2000 scale model has been shown to produce a superlinear speedup of the overall runtime when tested on several different parallel computing systems (Migliore et al., 2006). Using the principles detailed in that conversion, the more recent 1:20 scale dentate gyrus model (Dyhrfjeld-Johnsen et al., 2007; Morgan and Soltesz, 2008) was translated for compatibility with parallel NEURON. Because the granule cells in the control model network only fire sparsely as in the biological dentate gyrus (Morgan and Soltesz, 2008), the “injured” model, which contains several of the experimentally observed changes in epilepsy and displays epilepsy-related hyperactivity, was used in order to generate network activity for the parallel implementation. The general network topology of the dentate gyrus model is depicted in Figure 1A. The superlinear speedup from previous studies was also observed with the 1:20 scale model for up to the 90 processors tested, as shown in Figure 1B. The superlinear quality, instead of a purely linear result, is thought to be due to the more efficient use of a processor's memory (Migliore et al., 2006). The use of 90 processors decreased the overall runtime from 11.0 h to 6.8 min for 300 ms of model network activity. The granule cell activity for parallel model simulations is shown in Figure 1C. The model network connectivity and activity were identical for all parallel simulations regardless of the number of processors used.

**Figure 2:**
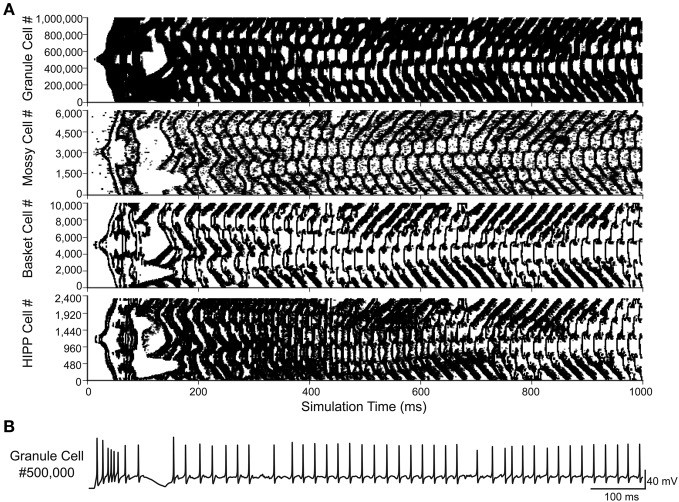
**Full-scale dentate gyrus simulation. (A)** Activity for each of the four cell types in the 80% injured model. Each dot represents a single spike. **(B)** Voltage trace from the simulation shown in **(A)** for granule cell #500,000.

The conversion to a parallel implementation makes the construction of a full-scale dentate gyrus computational model feasible. The increased availability of computational resources provided by parallel computing addresses the two main limiting factors to model network size: runtime and memory capacity. To construct the full-scale model, the number of cells and the connectivity of the 1:20 scale model were modified to be in agreement with the previous dentate gyrus structural model (Dyhrfjeld-Johnsen et al., 2007). The full-scale model contained over 1,000,000 cells and over 470,000,000 connections when simulated with the 80% injured model. The full-scale simulation shown in Figure 2 was performed on 150 processors, required ~220 GB of RAM, and was completed in 11.1 h. The reverberating network activity seen in previous scaled down injured models is observed in the full-scale network, shown for each cell type in Figure 2A. The granule cell activity spreads throughout the network and persists for the entire simulation time of 1 s. A voltage trace recorded from a granule cell demonstrates the realistic firing pattern of the model granule cells, shown in Figure 2B. The model, as explained above, represents a dentate gyrus from the epileptic rat brain with 80% hilar cell loss and heavy mossy fiber sprouting. The model depicted in Figure 2, while data-driven, still omits many of the changes known to occur in epilepsy, with hilar interneuron axon sprouting as one example (Zhang et al., 2009), as well as several other features, e.g., some interneuronal subtypes, gap junctions, short-term plasticity, etc. These features will need to be incorporated into the model in the future.

### Creating variability in dendritic morphology

Due to the additional computational resources provided by parallel computing, the complexity of the dentate gyrus model can be expanded just as with the network size. The single cell models are one of the previously simplified aspects of the model that can be improved, such as the single granule cell model. The current granule cell model [introduced in Santhakumar et al. (2005) for the 1:2000 model and also used in Dyhrfjeld-Johnsen et al. (2007) for the 1:20 model] contains nine cylindrical compartments with the diameter of the dendrites constant throughout the dendrites, based on a previous computational model (Aradi and Holmes, 1999). In addition, the morphology and biophysics are equivalent for every granule cell in the network, so that the 1:20 scale model contains 50,000 identical simplified granule cells. The introduction of realistic morphology and cell-to-cell variability would greatly improve the complexity of the network model and can be achieved using two freely available software tools: L-Measure (Scorcioni et al., 2008) and L-Neuron (Ascoli and Krichmar, 2000).

**Figure 3:**
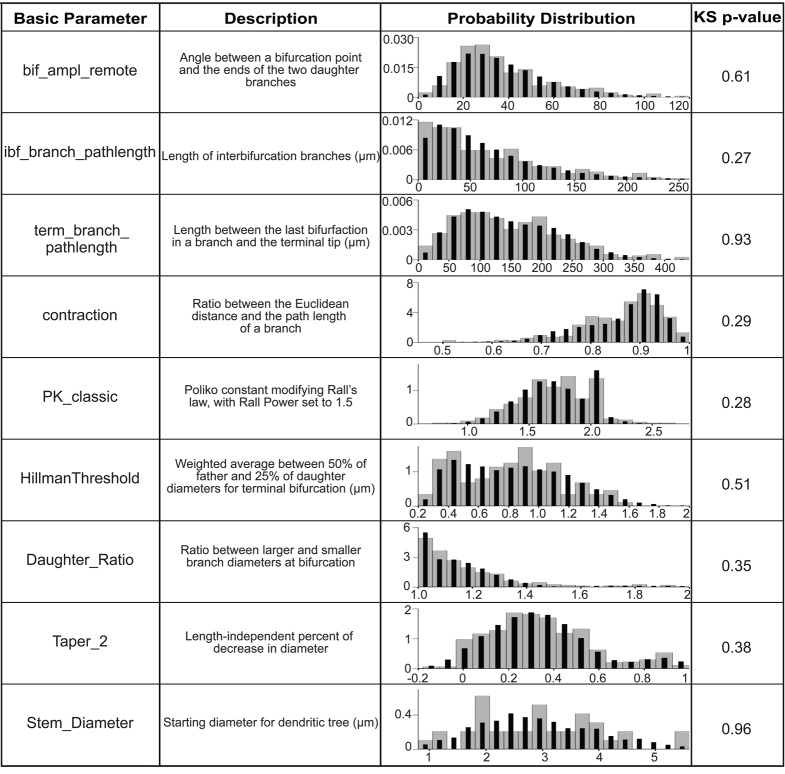
**Basic parameter distributions are matched in order to generate virtual granule cells.** The basic parameters extracted from L-Measure and incorporated into L-Neuron are listed with their definitions. For more information see the L-Measure website listed in Materials and Methods. The probability distributions are plotted as probability density versus the basic parameter value for experimental reconstructions (gray bars) and 10,000 generated virtual neurons (black bars), and the *p*-value for the two-sample Kolmogorov–Smirnov test for the overlap of these distributions is shown.

The general strategy for virtual neuron generation was to use morphological parameters extracted from granule cell reconstructions to create realistic virtual neurons that are as much as possible indistinguishable from the reconstructed cells. Basic morphological parameters required by the L-Neuron dendritic growth algorithm were extracted from granule cell reconstructions using L-Measure and are listed in Figure 3. The extracted parameter distributions were incorporated into L-Neuron to generate virtual dendritic trees with the same basic parameters as the experimental reconstructions. There was no significant difference between real and virtual granule cell basic parameter distributions (Figure 3; *p* > 0.05, two-sample Kolmogorov–Smirnov test). The L-Neuron program thus creates granule cell dendritic trees with similar basic parameters to those seen in granule cells in the biological dentate gyrus. However, the basic parameter distributions used in L-Neuron have a high degree of variability, and it has been noted by previous studies (Ascoli et al., 2001; Donohue et al., 2002) that virtual dendrites generated with L-Neuron have an excessive degree of variability when compared with sample dendrites. Accordingly, generated dendrites will not necessarily be biologically realistic nor produce a representative population. To counteract the excessive variability, the high degree of variability in L-Neuron inputs is still allowed, but the outputs are filtered to select for only those morphologies that are representative of the experimental reconstructions. A population can then be constructed from these biologically realistic morphologies that have a similar degree of variability as the sample set of reconstructions.

**Figure 4:**
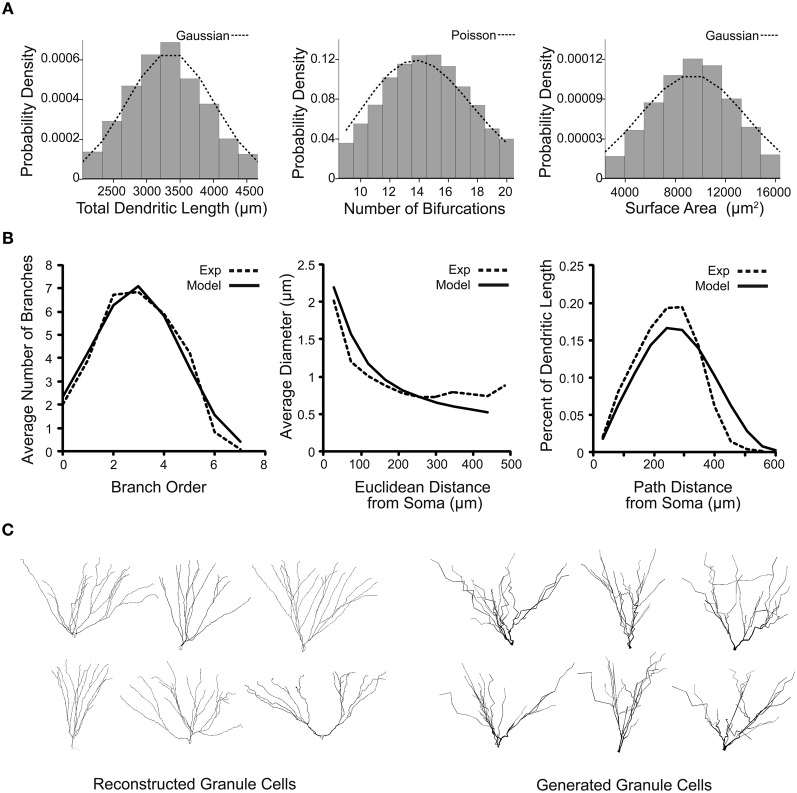
**Emergent parameter distributions demonstrate the creation of variable and realistic virtual granule cells of the dentate gyrus. (A)** Probability distributions for several scalar emergent parameters of 100,000 generated granule cells. Distributions for total dendritic length, number of bifurcations, and surface area are shown with their ideal respective truncated Gaussian and Poisson probability density functions. **(B)** Distribution emergent parameters for real (dashed line) and 10,000 generated (solid line) neurons. **(C)** Sample reconstructed and generated dentate granule neurons, visualized in NEURON.

The generated granule cells were filtered based on scalar emergent parameters that are not used in the dendritic growth algorithm, such as the total dendritic length of the cell. Constraints involving variability up to two standard deviations for most emergent parameters allowed for values beyond those measured from the sample reconstructions, but restricted generated neurons to only those that have biologically realistic values. The virtual granule cells were then chosen to match a Gaussian or Poisson distribution for the total dendritic length, number of bifurcations, and surface area, shown in Figure 4A, to create a representative and distributed population. The virtual granule cell population and granule cell reconstructions were also compared using distribution emergent parameters that examine the relationship between two parameters. The distribution emergent parameters shown in Figure 4B demonstrate that the generated granule cells are nearly indistinguishable from the reconstructed cells. This method of constraining and selecting virtual dendrites allows a small set of experimental reconstructions to be amplified to create a realistic and distributed population of granule cells. A sample of real and virtual granule cells is shown in Figure 4C. The dendritic trees can be improved by adding tropism to increase the curvature and smoothness of the dendrites, but the overall structures for real and generated neurons are statistically indistinguishable.

## Discussion

The model development presented in this study reflects a significant advancement in bridging the gap between the biological dentate gyrus and the dentate gyrus network model. The speedup conferred by parallel computing makes the 1:20 scale model more accessible for use with even a small computing cluster by reducing the time required for simulations, and the additional computational resources enable the model to be implemented at full scale. These full-scale simulations are advantageous in that they remove the scaling adjustments made for smaller models, and values determined experimentally can now be directly incorporated into the model. The computational model thus becomes a more effective complement to experimental techniques.

The additional computational resources also allow for the complexity of the network to be increased to provide a greater level of accuracy, including at the level of single cells. The inherent cooperation between morphological analysis with L-Measure and generation with L-Neuron provides a means by which to amplify a small sample of reconstructed cells into a distributed virtual population. The availability of detailed reconstructions is ever-growing, such as through the Neuromorpho.org database (Ascoli et al., 2007), so the tools and principles used in this study can be applied to other cell types within the dentate gyrus. The potential covariance of morphological parameters, however, is not considered in this study. Similarly, the inter-dependence of physiological parameters will also need to be addressed, especially with a functional model, given previous insights (Golowasch et al., 2002). The introduction of realistic morphology expands the network from a two-dimensional structure that is dominated by a linear strip to a three-dimensional model that allows for cells to be distributed in space and throughout layers. This ability gives rise to the possibility of making more direct comparisons to the experimental literature, through concepts such as taking a virtual slice of the model.

The incorporation of realistic morphology can be used in concert with experimentation on the passive and active properties of dentate gyrus neurons to create accurate and variable neurons. Recent studies using a combination of experimental methods and computational modeling have described the dynamics of action potential initiation (Schmidt-Hieber and Bischofberger, 2010) and dendritic integration (Schmidt-Hieber et al., 2007; Krueppel et al., 2011) for dentate granule cells. Generated functional models will need to possess the documented characteristics of granule cells in these studies, such as axonal action potential initiation and a similar level of dendritic attenuation of backpropagating action potentials. In addition, the passive and active properties (e.g., resting membrane potential, action potential threshold, etc.) will be compared to literature values, similar to the development of our previous single cell model (Santhakumar et al., 2005). The improvement of single cell models is not reserved only to principal cells, as the properties of fast-spiking basket cells determined through experiment and modeling (Hu et al., 2010; Norenberg et al., 2010), for example, can be incorporated into a more accurate basket cell model. Through these improvements and the resources conferred by parallel NEURON, the computational model of the rat dentate gyrus can reflect a previously unattainable size and accuracy in order to approach a more complete model of the biological dentate gyrus.

### Conflict of interest statement

The authors declare that the research was conducted in the absence of any commercial or financial relationships that could be construed as a potential conflict of interest.
